# Supplementary material for: Optomechanical crystals for spatial sensing of submicron sized particles
Source: Sci Rep. 2021 Apr 9;11:7829. doi: 10.1038/s41598-021-87558-4 (PMC8035185; doi:10.1038/s41598-021-87558-4)
Supplement: Supplementary file 1 — Supplementary Information [file 41598_2021_87558_MOESM1_ESM.docx]

Supplementary Info: Optomechanical crystals for spatial sensing of submicron sized analytes

D. Navarro-Urrios^1,2*^, E. Kang^3^, P. Xiao^2^, M. F. Colombano^1,2^, G. Arregui^2^, B. Graczykowski^3,4^ N. E. Capuj^5,6^, M. Sledzinska^2^, C. M. Sotomayor-Torres^2,7^, G. Fytas^3*^

^1^ MIND-IN2UB, Departament d'Enginyeria Electrònica i Biomèdica, Facultat de Física, Universitat de Barcelona, Martí i Franquès 1, 08028 Barcelona, Spain

^2^ Catalan Institute of Nanoscience and Nanotechnology (ICN2), CSIC and BIST, Campus UAB, Bellaterra, 08193 Barcelona, Spain

^3^ Max Planck Institute for Polymer Research, Ackermannweg 10, 55128 Mainz, Germany

^4^ Faculty of Physics, Adam Mickiewicz University, Umultowska 85, 61614 Poznan, Poland

^5^ Depto. Física, Universidad de La Laguna, 38200 San Cristóbal de La Laguna, Spain

^6^ Instituto Universitario de Materiales y Nanotecnología, Universidad de La Laguna, 38071 Santa Cruz de Tenerife, Spain

^7^ Catalan Institute for Research and Advances Studies ICREA, 08010 Barcelona, Spain

**S1. Band diagrams of the nominal unit cell**

Figure 1S displays photonic and phononic band diagrams (left and right panels, respectively) corresponding to the nominal design, whose geometrical parameters have been written in terms of *a* and Γ. Frequencies ω are given with dimensionless units, i.e., they are divided by *c/a*, where *c* is either the velocity of light in vacuum for electromagnetic waves or the transverse speed of sound in silicon in [100] direction (*c*_t_=5844 m/s). In the case of the photonic dispersion, we have only considered two relevant TE-polarized (*y*-direction) bands that define a TE photonic gap between ω=0.2 and ω=0.25. In the case of phononic dispersion, we have considered the lowest energy band displaying an even-even symmetric band with respect to the *xy* and *xz* planes. The modes that verify these conditions are the “pinch” mechanical modes represented in Figure 1 of the main text.

We have also studied the dependence of the energy of the Bloch modes at the x-point with Γ. Regarding the photonic bands, optical modes are drawn up in energy by decreasing Γ. Thus, if the cavity region between the mirrors is constructed so that the pitch is gradually reduced towards the center, cavity optical modes are expected to appear owning frequencies slightly higher than that of the edge of the lower band. On the contrary, the “pinch” band is pushed down by decreasing Γ, so that the cavity mechanical modes would have slightly lower energies than that of the edge of the band.

The geometrical parameters of the fabricated unit cell have been rescaled to the values reported in the main text so that the spectral range of the tunable laser (around 200 THz) covers the frequency region swept by the lower photonic band edge when Γ is varied between 1 and 0.9.


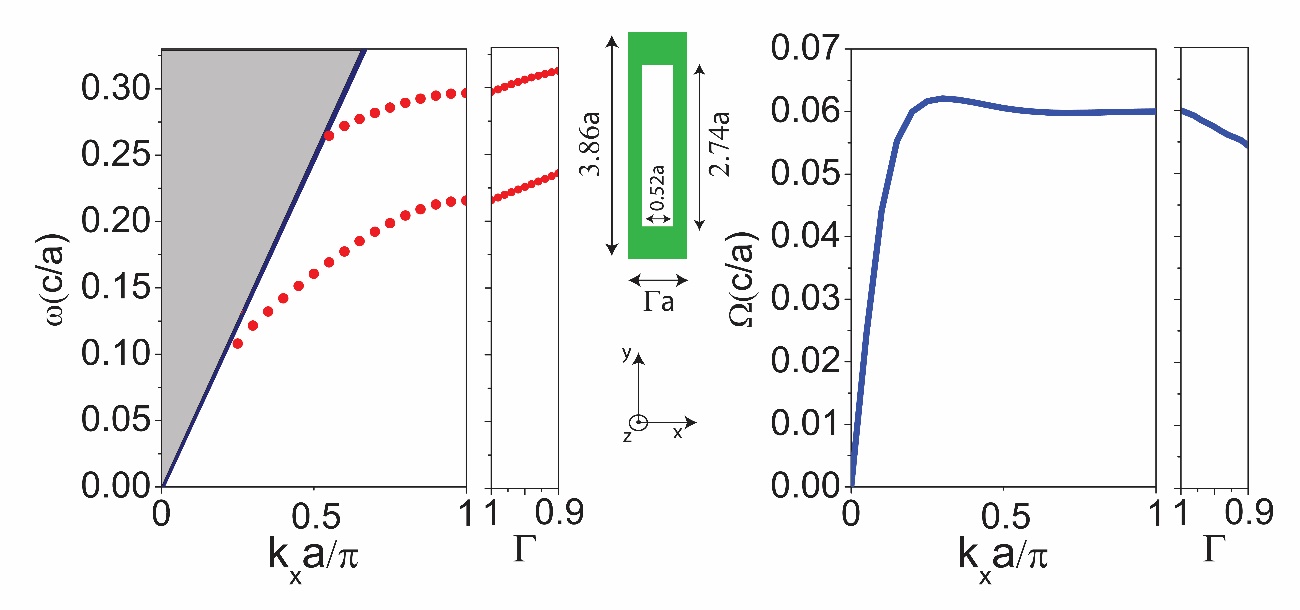


**Figure S1.** Photonic (left) and phononic (right) normalized band diagrams of the unit cell depicted in green. We have only considered the relevant bands for the purpose of this work, i.e., TE-polarized modes and “pinch” modes for the photonic and phononic calculations, respectively.

**S2. OM coupling calculations**

Single-particle Optomechanical (OM) coupling rates (*g*_O_) between optical and mechanical modes are estimated by taking into account both photo-elastic (PE) and moving-interface (MI) effects ^1^,^2^,^3^. The PE effect is a result of the acoustic strain within bulk silicon while the MI mechanism comes from the dielectric permittivity variation at the boundaries associated with the deformation.

The calculation of the MI coupling coefficient *g*_MI_ is performed using the integral given by Johnson et al. ^1^;

(S1)

where **Q** is the normalized displacement (max{|**Q**|}=1), $\hat{\mathbf{n}}$ is the normal at the boundary (pointing outward), **E** is the electric field and **D** the electric displacement field. $\text{ε}$ is the dielectric permittivity, *Δε=ε*_silicon_*- ε*_air_ , *Δε^−1^=ε^−1^*_silicon_*- ε^−1^*_air_. *λ*_r_ is the optical resonance wavelength, *c* is the speed of light in vacuum, is the reduced Planck constant, *m*_eff_ is the effective mass of the mechanical mode and *Ω*_m_ is the mechanical mode eigenfrequency, so that is the zero-point motion of the resonator.

A similar result can be derived for the PE contribution ^2^,^3^:

(S2)

where *δε_ij_=ε*_air_ *n^4^p_ijkl_S_kl_*, being *p_ijkl_* the PE tensor components, *n* the refractive index of silicon, and *S_kl_* the strain tensor components.

The addition of both contributions results in the overall single-particle OM coupling rate:

$g_{O}=g_{MI}+g_{PE}$ (S3)

**S3. Design of the Optomechanical crystal**

The defect used here consists of an odd number of cells, with the pitch reduced quadratically towards the center down to a value equal to Γ*a*. On both sides of the cavity region the nominal cell is repeated over 10 times, thus acting as an effective mirror for optical and mechanical cavity modes. FEM simulations have verified that adding more mirror cells does not improve further the quality factors of the cavity modes.

The cavity region has been distributed over a total number of cells denoted by *N*, which determines the sensing area (*S*). However, the defect region cannot be distributed over an arbitrary large number of cells. Indeed, if the various cells composing the defect region are not different enough among them, a pinch mode can involve the deformation of several cells. In terms of sensor design this implies that spatial resolution would be lost at the expense of increasing *S*. Thus, a compromise has been found in terms of having the largest number of cells composing the cavity region, i.e., the largest *S* value, while also verifying that: i) pinch modes do not involve more than two cells and ii) the frequency difference between two adjacent modes is larger than the experimental mechanical linewidth (about 1 MHz). In Figure S2 we show the results of computing the normalized g_O_ for different realizations of an OMC in which the number of cells composing the cavity region has been increased from *N*=11 to *N*=31. In order to resemble realistic RF spectra we have associated to each mode a Lorentzian distribution owning a linewidth equal to that required, finally adding up the contribution of all modes.

As expected, the pinch modes appear in the region between the bad edge values for Γ=0.9 and Γ =1 (left and right dashed areas, respectively) and their number roughly scales with *N*. The deformation profiles of the modes are localized more towards the centre as their frequency decrease, in agreement with what extracted from the study of the dependence of the band edge energy with Γ. Up to *N*=27 all the computed modes involve at most two cells and display a frequency separation that in average fulfils the requirements. This starts to be no longer valid for larger *N* values. This can be observed in the lowest panel of Figure S2, where in the lower frequency region the discrimination of distinct RF peaks would be no longer straightforward.

On the basis of these simulations, the cavity region was chosen to be distributed over 27 central holes, whose sensing area is about 12 μm^2^.


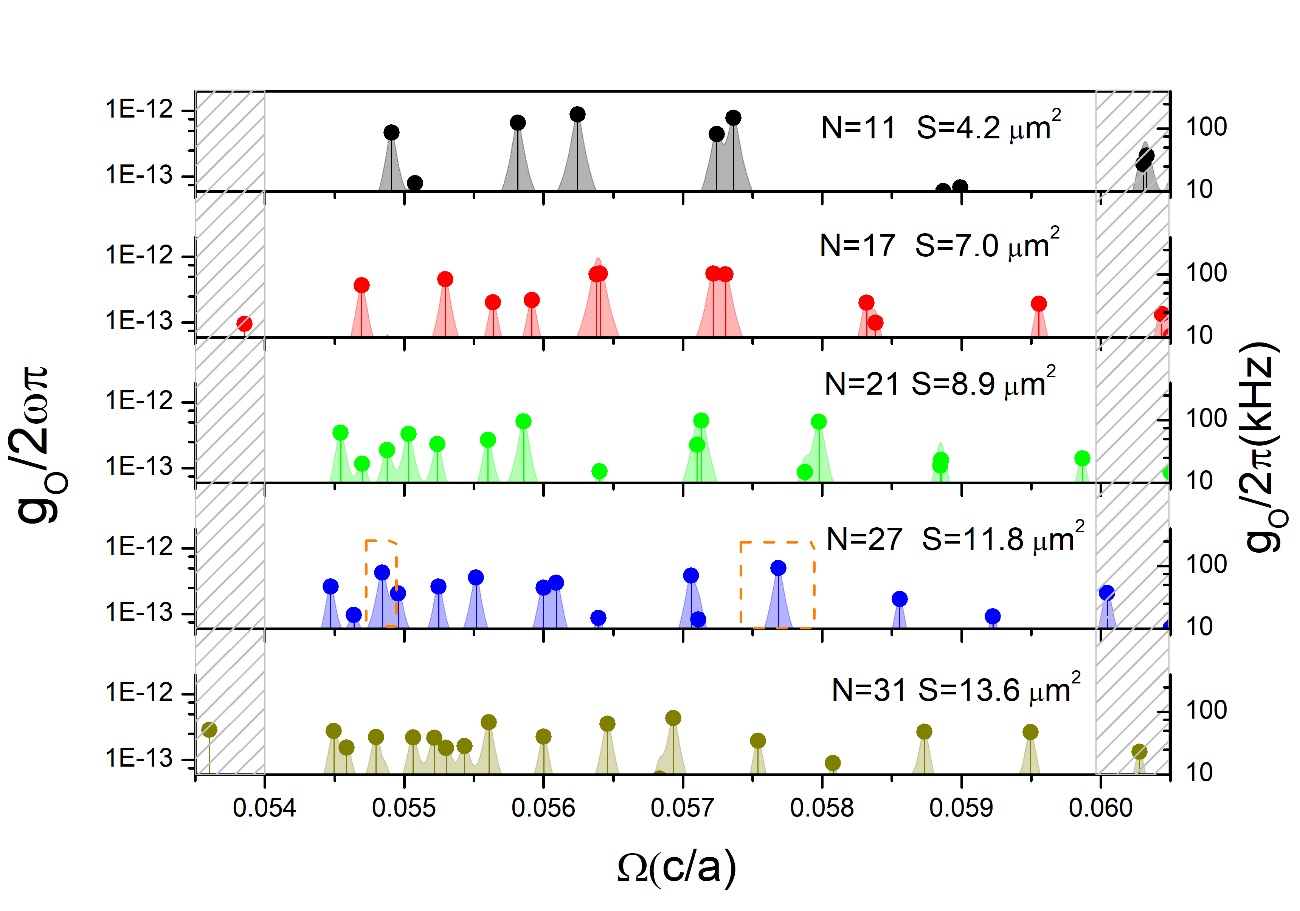


**Figure S2.** Normalized optomechanical coupling rates for different values of the total number of cells building the cavity region. The right vertical axis represents *g*_O_/2π considering a geometry scaled so that the optical mode appears around 180 THz. The dashed regions define the allowed region for cavity modes, which lies between the band edge values for Γ=0.9 (left) and Γ =1 (right). The highlighted peaks are associated to the couples of optical and mechanical modes considered for the illustrations of Fig. S4.

In Figure S3 we show the computed optical modes as a function of N while keeping fixed Γ=0.9. The OMC has been scaled so that the modes appear above 180 THz, thus falling in the spectral range covered by the tunable laser. It is also observed that the confined modes appear in the region between the bad edge values for Γ=0.9 and Γ =1.

**Figure S3.** Optical modes as a function of the number of cells building the cavity region. The coloured area illustrates the spectral range covered by the tunable laser.

To illustrate better the optomechanical properties of this particular OMCs in Figure S4 we represent the different spatial contributions relevant for the calculation of *g*_O_ for the particular realization having *N*=27 and Γ=0.9. In particular, we have focused on the optical mode in Figures S4a and S4e, and the mechanical modes in Figures S4b and S4f, which correspond to those highlighted in Figure S2 with dashed orange boxes. The optical mode is one appearing close to the band edge and thus extends over the whole cavity region. As a consequence, *g*_O_/2π takes values (greater than 10 kHz considering a geometry scaled so that the optical mode appears at 180 THz) for the whole family of pinch cavity modes, as also illustrated in Figure S2. The mechanical mode appearing in the high (low) frequency region Ω(*a/c*)= 0.577 (0.548) is localized away from (close to) the center. In addition, both involve the oscillation of just a couple of adjacent cells, which is in agreement with what discussed above. This is also evidenced in Figures S4c and S4g, and Figures S4d and S4h, where we illustrate the PE volume density (the integrand of Eq. S2) and the MI surface density (the integrand of Eq. S1), respectively. Indeed, the volume contributing significantly to *g*_O_ is reduced to the coloured regions. It is also worth mentioning that, in the pinch modes of the current study, *g*_O_ is dominated by the MI contribution given that the PE contribution is an order of magnitude smaller.


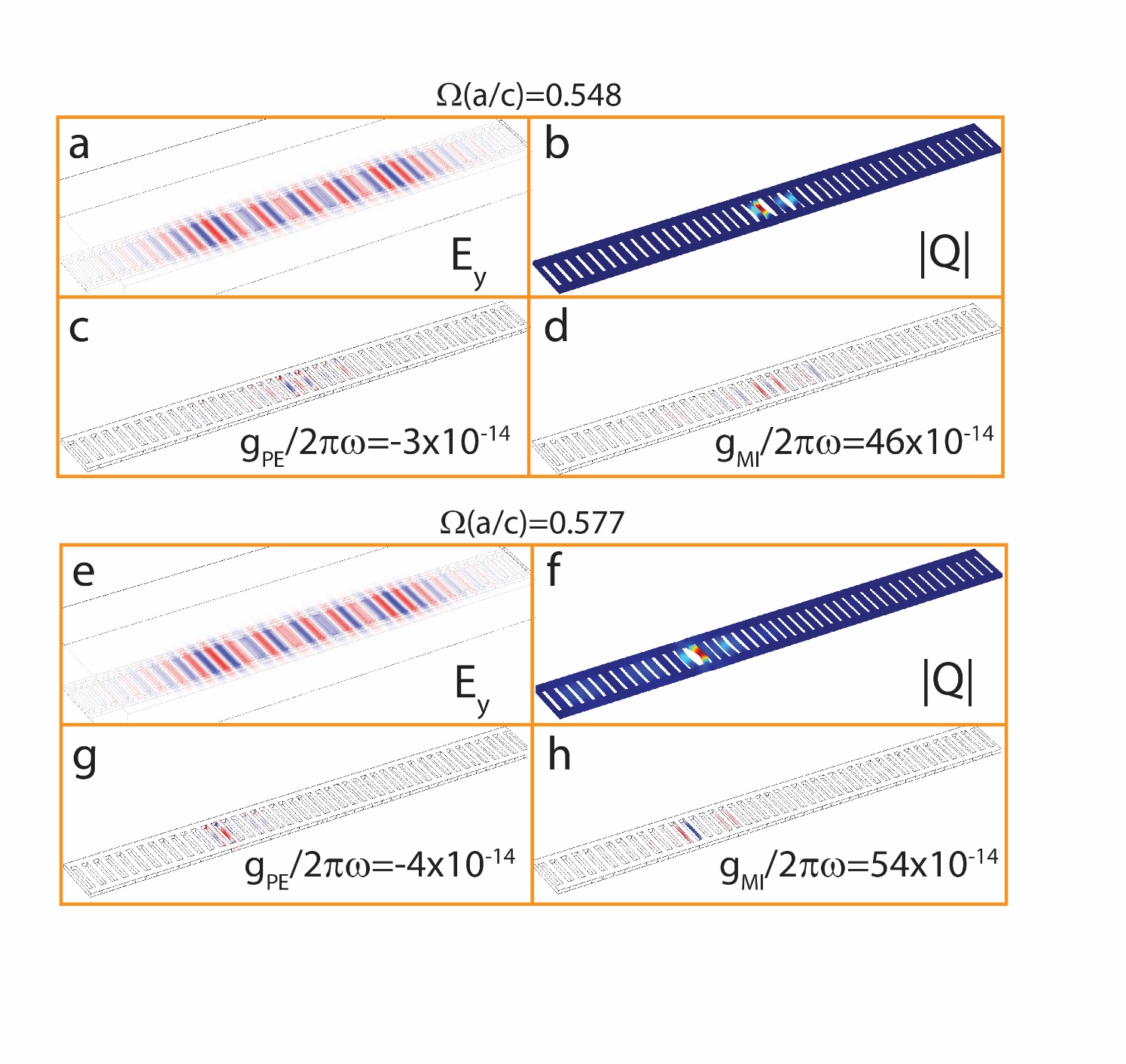


**Figure S4. Optical and mechanical modes of the Optomechanical Crystal cavity with N=27 and G=0.9 cells considering mechanical modes at Ω(a/c)=0.548 and 0.577 (top and bottom panels, respectively).** a) and e) Normalized optical E_y_ field of the optical mode supported by the OM crystal used for the calculations reported in Fig. S2. b) and f) Normalized mechanical displacement field |Q| of the pinch mechanical mode. c) and g) Normalized volumetric density of the integrand of the integrand in Eq. S2, showing the contributions to g_PE_. d) and h) Normalized surface density of the integrand in Eq. S1, showing the contributions to g_MI_.

**S4. Fabrication of the Optomechanical Crystal**

The structures were fabricated in Silicon-on-Insulator (SOI) samples with a top silicon layer thickness of 250 nm (resistivity *ρ* ~1–10 Ohm.cm, p-doping of ~1×10^15^ cm^−3^) and a buried oxide layer thickness of 2 μm. The OMC cavities fabrication process was based on electron beam direct writing on a coated 170 nm of CSAR resist layer. The electron beam exposure was optimized with an acceleration voltage of 10 KeV and an aperture size of 30 μm with a Raith150 tool. After developing, the resist patterns were transferred into the SOI samples by inductively coupled plasma reactive ion etching. Finally, the silicon dioxide under the membranes was removed by using a HF bath.

**S5. Influence of particle elastic properties on spectral position of affected pinch modes**

We have performed FEM simulations using the geometry imported from the SEM micrograph showed in Figure 2 in which we have modified the Young modulus and density of the particle. We verified that the affected pinch mode does not change further its frequency or spatial distribution in a wide range of input values around those of bulk SiO_2_ (left and central panels of Figure S5). We have also shifted down the particle position along the crossbar (right panel of Figure S5), verifying that the mode is only slightly altered until when the particle starts loosing contact with the crossbar. In that situation the crossbar is released and the original mode displayed by the OMC without considering the particle is recovered.


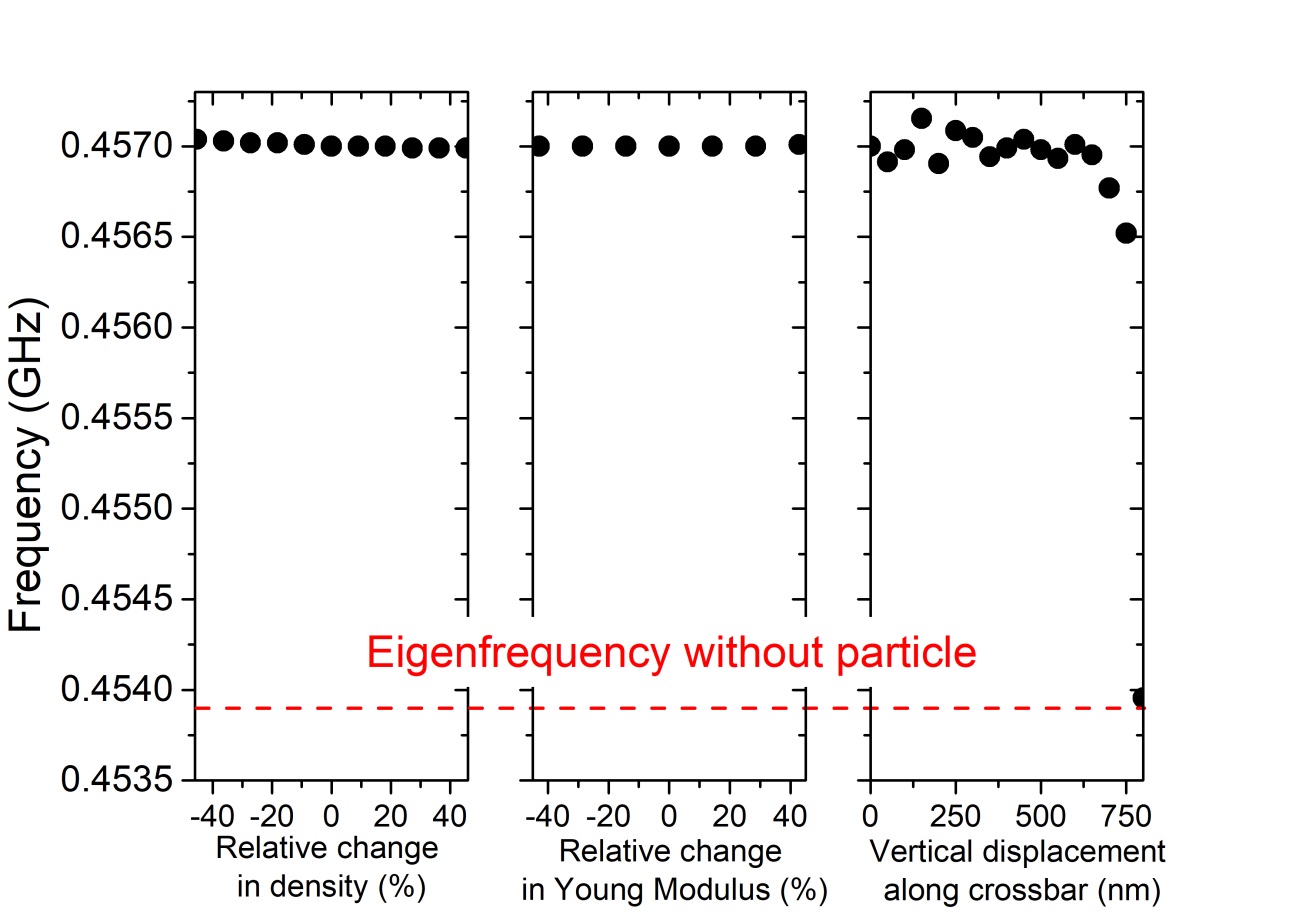


**Figure S5.** Dependence of eigenfrequency of the mechanical modes highlighted in Figure 2 of the main text with respect to relative changes in particle density (left panel) and Young modulus (central panel) and vertical displacement along the crossbar (right panel). The horizontal dashed line represents the eigenfrequency of the original mode without considering the particle on top of the OMC.

**S6. Pinch like mechanical modes of the fabricated Optomechanical Crystal involving the oscillation of the submicron particle**

The presence of the submicron particles modifies the pinch mechanical mode spectrum as reported in Figures 2 and 3 of the main text. In addition, there are other worth mentioning modifications of the mechanical spectra. The first one is the onset of pinch-like modes that involve the collective oscillation of the particle and the bar in contact with it. These latter modes appear at significantly lower frequencies than those plotted in Figure 2, given that their effective mass is about a factor of two larger (Figure S6).


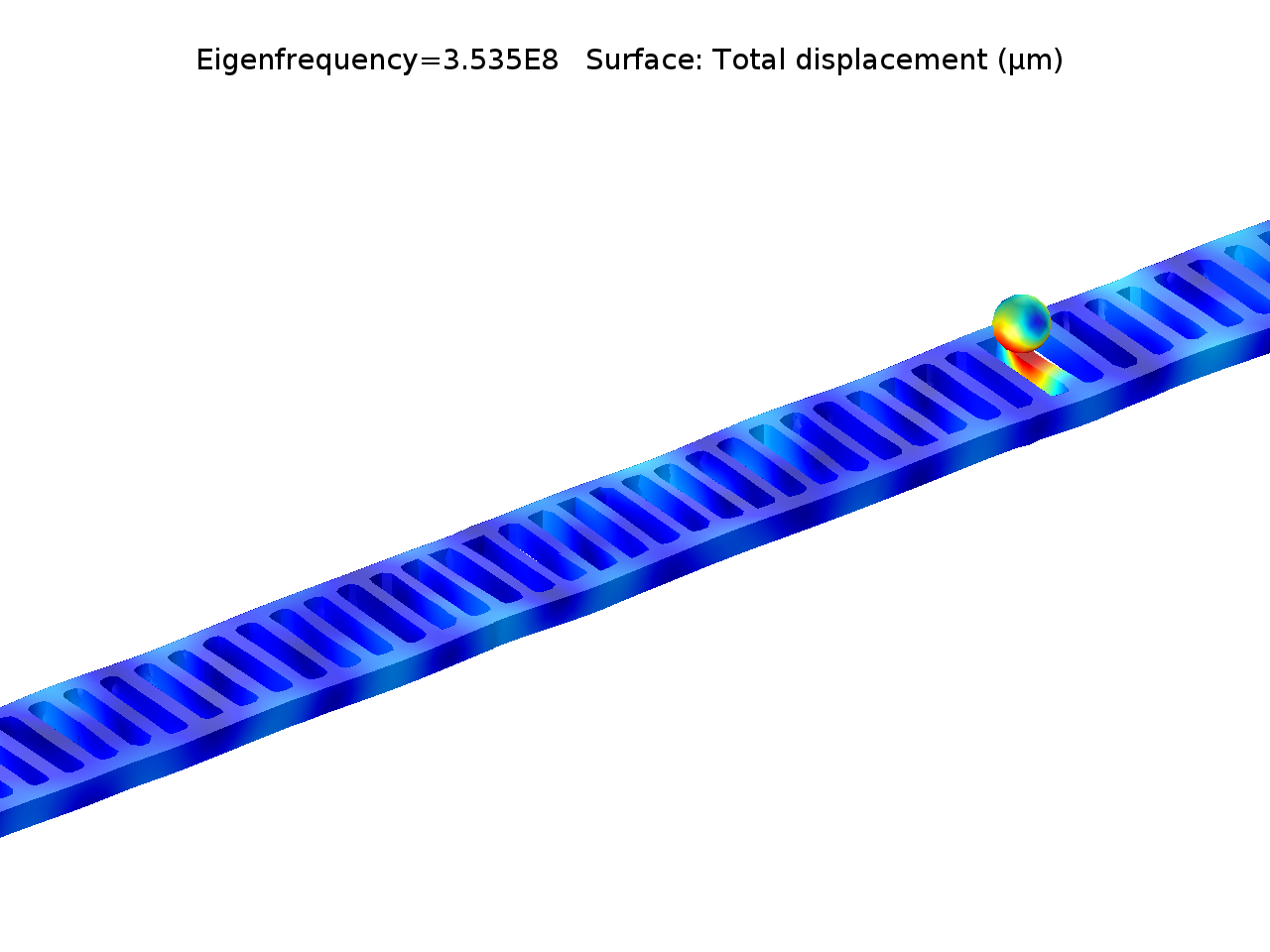


**Figure S6.** Pinch-like mode involving the collective oscillation of the particle and the bar in contact with it. This mode appears at significantly lower frequencies (0.35 GHz) than those plotted in Figure 2 of the main text, given that their effective mass is about a factor of two larger (about 0.2 pg).

Mechanical modes involving solely the deformation of the silica particle (*d*=500 nm) start appearing at much higher frequencies (few GHz, see Figure S8), in consistence to what obtained on an isolated particle of the same size.


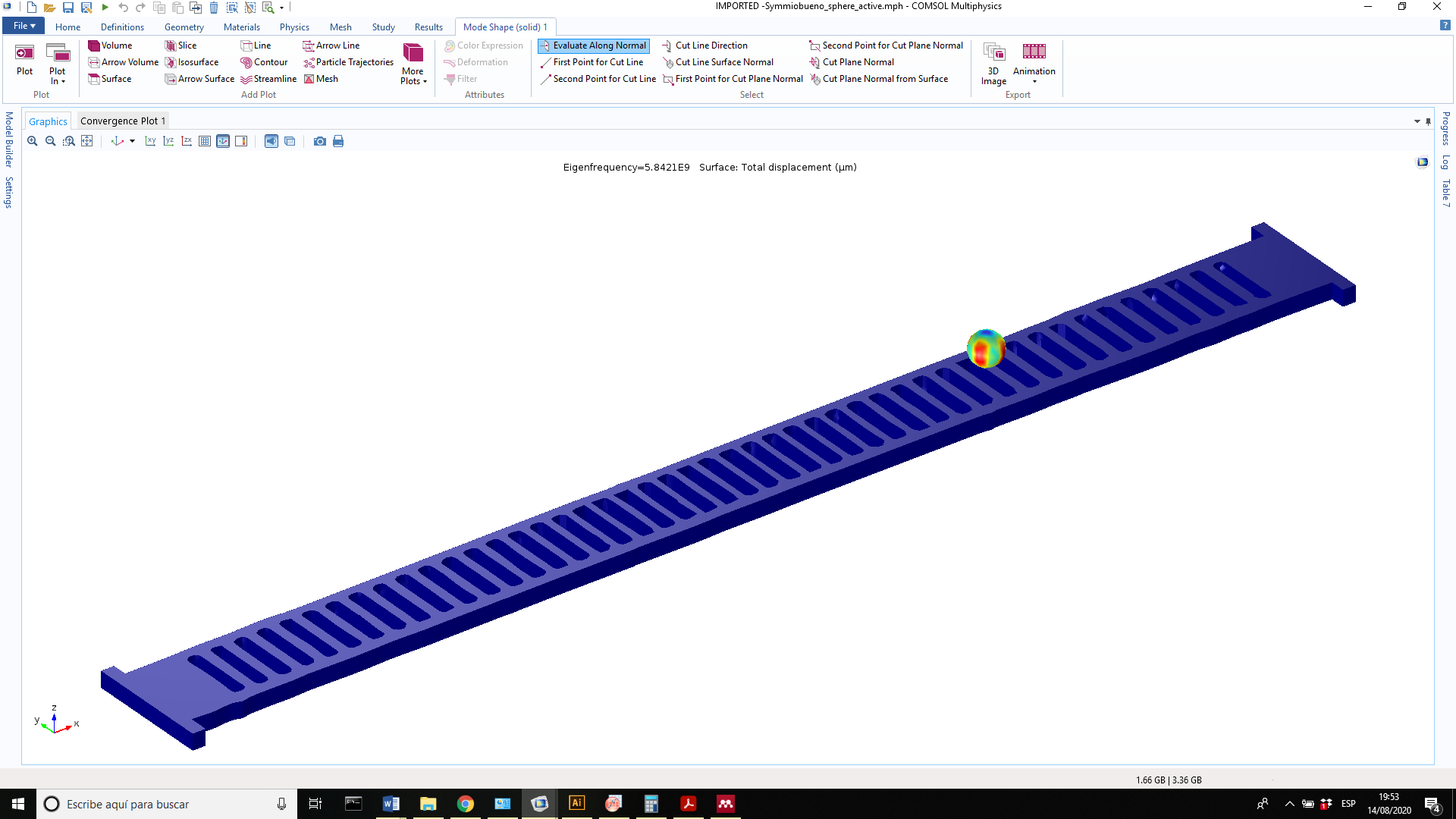


**Figure S7**. Mechanical mode involving solely the oscillation of the spherical particle. The calculated frequency of this particular mode is about 5.8 GHz.

We have also modified the Young modulus of the particle and its position along the crossbar and verified that the modified mode does not change further its frequency or spatial distribution. The previous statement holds unless there are mechanical eigenfrequencies of the isolated particle that are similar to those of the original pinch modes, in which case the modes hybridize similarly to what experimentally demonstrated in Ref. ^4^. In Figure S8 we show that this is in fact the case if the elastic properties and the size of the spherical particle are modified to bring down its oscillation frequency to the frequency range of the pinch modes, i.e., to about 0.48 GHz.


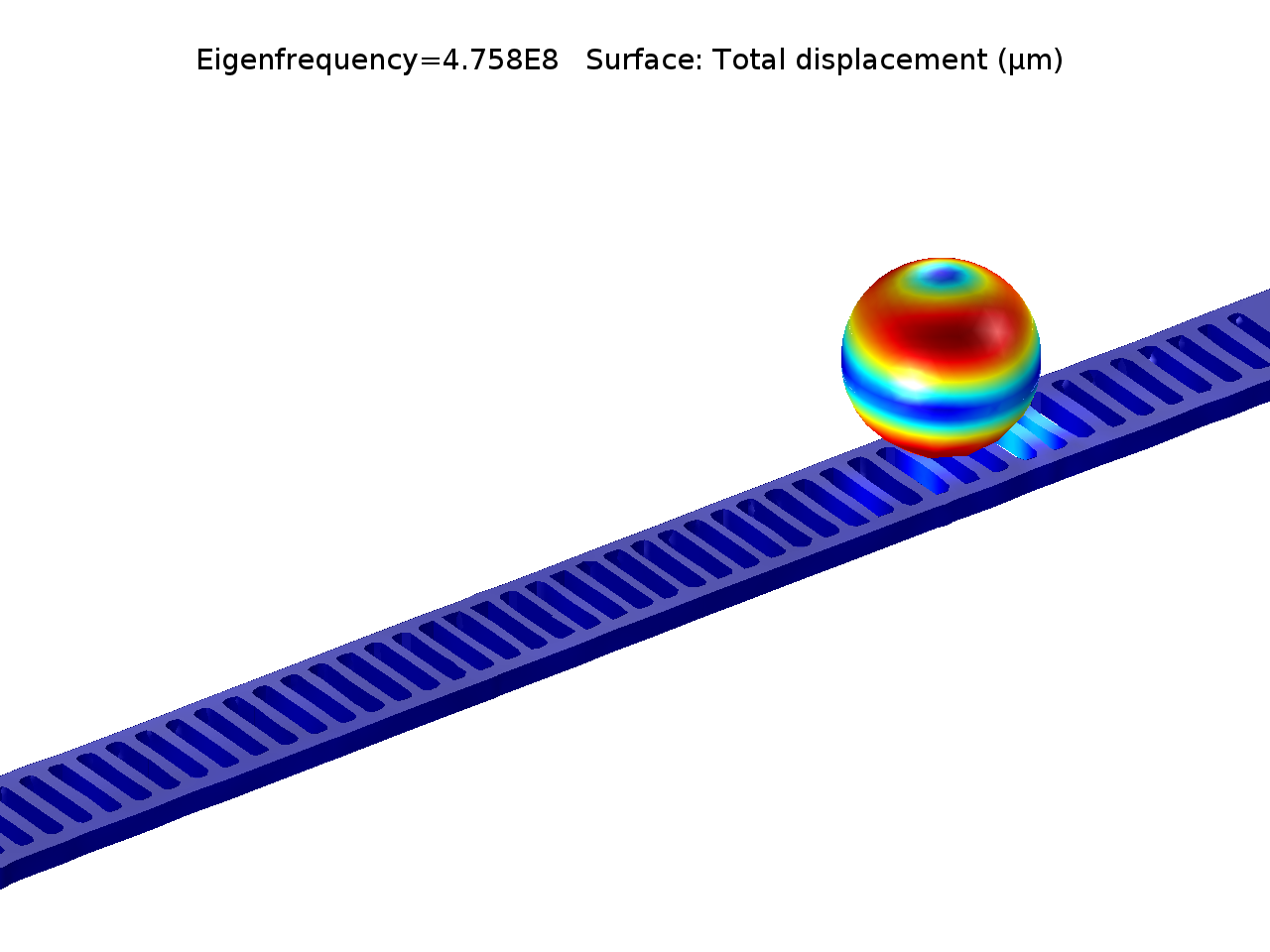


**Figure S8.** Pinch-like mode in which the natural mode of the particle hybridizes with a pinch mode of similar frequency. The elastic properties and the size of the spherical particle have been modified to bring down its oscillation frequency to about 0.48 GHz, but its positioning is the same as in the case considered in Fig. 2 and Fig. S6.

**S7. Eigenvibrations of the Silica Nanoparticles**

The eigenmode vibration spectrum of the present silica nanoparticles (diameter *d* = 495 ± 16 nm recorded by spontaneous Brillouin light spectroscopy (BLS) ^5^ is shown in Figure S9 (anti-stokes side). The lowest frequency quadrupolar (1,2) mode appears at 3.67GHz is much higher than the OMC resonance frequency. Based on the *f*(1,2) = 0.85*c*_t_*/d,* the transverse speed of sound is *c_t_* =2140 m/s [5].

**
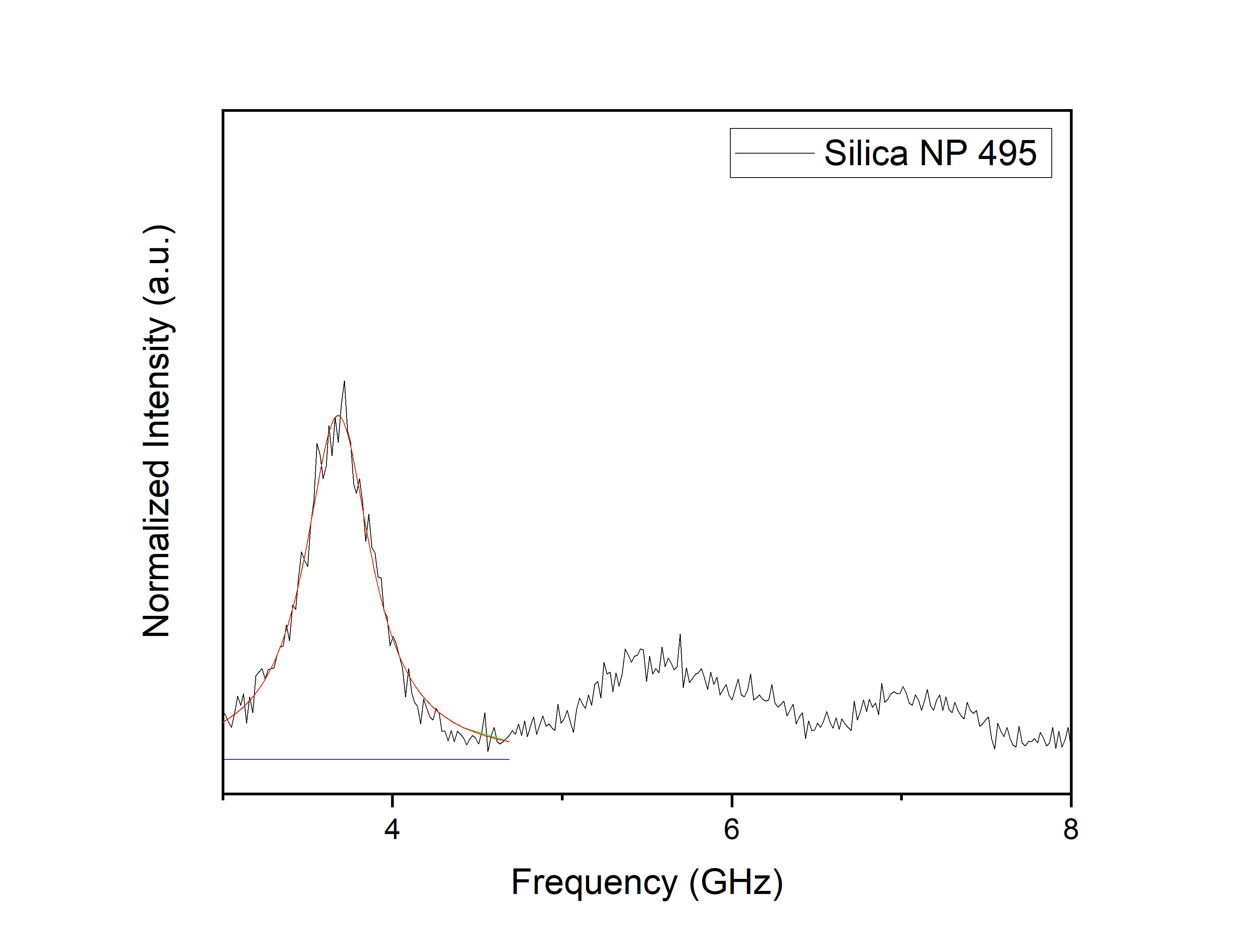
**

**Figure S9.** BLS eigenmode spectrum (anti-Stokes side) of spherical silica nanoparticles with diameter *d* = 495 ± 16 nm.

**S8. Comparison of SEM micrographs under zenithal and tilted configurations.**

In Fig. S10 we show that the particles that appear below the OMC in an SEM image taken from the top (top panel) are lying on the substrate and, therefore, not in contact with the OMC. This becomes apparent when tilting the sample (bottom panel).


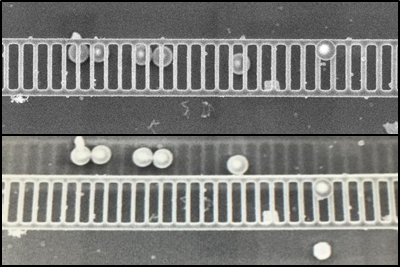


**Figure S10.** SEM micrographs of the characterized OMCs with one submicrometer particle on top. The top and bottom panels illustrate a zenithal and a tilted view, respectively.

In Fig. S11 we show a zoomed SEM image of the OMC in a tilted configuration, where it is possible to see that the walls are slightly undulatory and probably reentrant. In the FEM simulations we assume that the projected section, which is what we get from a SEM top view, is maintained over the whole thickness of the geometry, which leads to overestimating the volume of the nanobeam and probably to the observed differences between the simulated and experimental spectra.


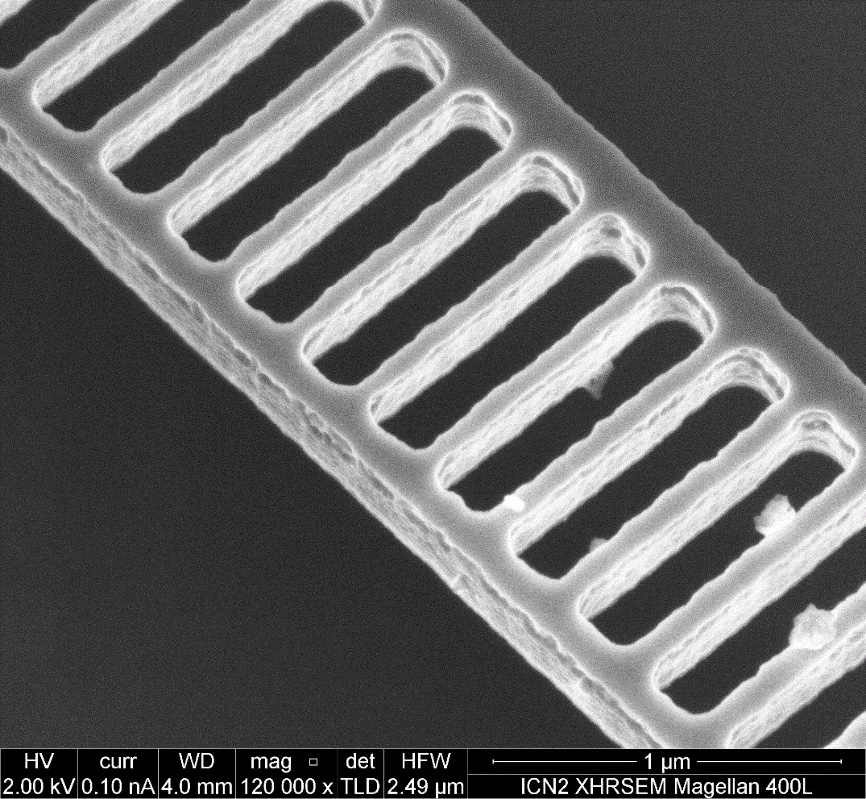


**Figure S11.** Large magnification SEM micrographs of the characterized OMCs under tilted configuration.

**S9. Comparison of mechanical spectra of clean samples (before and after having deposited the particles)**

**Figure S12.** Transduced mechanical modes in the frequency range of the pinch modes family for the case of the as-fabricated OMC (black) and the OMC after cleaning the last particle (red).

**S10. Q-factor of mechanical modes at large frequencies**

These structures have other families of mechanical modes with large enough OM coupling rates to be detected optically and placed in different frequency ranges. Those derive from other bands of the periodic phononic crystal. ^6^ In particular, there is a family of “breathing” mechanical modes around 2 GHz, which display significantly larger quality factors, about 770 (see Figure S13), than the “pinch” modes.

Fig. S13. RF spectrum in linear scale of a breathing mode of the OMC under study appearing at 2.15 GHz. The Q-factor is about 770.

**References**

(1) Johnson, S. G.; Ibanescu, M.; Skorobogatiy, M. A.; Weisberg, O.; Joannopoulos, J. D.; Fink, Y. Perturbation Theory for Maxwell’s Equations with Shifting Material Boundaries. *Phys. Rev. E* **2002**, *65* (6), 66611. https://doi.org/10.1103/PhysRevE.65.066611.

(2) Chan, J.; Safavi-Naeini, A. H.; Hill, J. T.; Meenehan, S.; Painter, O. Optimized Optomechanical Crystal Cavity with Acoustic Radiation Shield. *Appl. Phys. Lett.* **2012**, *101* (8), 81115. https://doi.org/10.1063/1.4747726.

(3) Pennec, Y.; Laude, V.; Papanikolaou, N.; Djafari-Rouhani, B.; Oudich, M.; Jallal, S. El; Beugnot, J. C.; Escalante, J. M.; Martínez, A. Modeling Light-Sound Interaction in Nanoscale Cavities and Waveguides. *Nanophotonics* **2014**, *3* (6), 413–440. https://doi.org/https://doi.org/10.1515/nanoph-2014-0004.

(4) Gil-Santos, E.; Ruz, J. J.; Malvar, O.; Favero, I.; Lemaître, A.; Kosaka, P. M.; García-López, S.; Calleja, M.; Tamayo, J. Optomechanical Detection of Vibration Modes of a Single Bacterium. *Nat. Nanotechnol.* **2020**, *15* (6), 469–474. https://doi.org/10.1038/s41565-020-0672-y.

(5) Still, T.; Mattarelli, M.; Kiefer, D.; Fytas, G.; Montagna, M. Eigenvibrations of Submicrometer Colloidal Spheres. *J. Phys. Chem. Lett.* **2010**, *1* (16), 2440–2444. https://doi.org/10.1021/jz100774b.

(6) Eichenfield, M.; Chan, J.; Camacho, R. M.; Vahala, K. J.; Painter, O. Optomechanical Crystals. *Nature* **2009**, *462*, 78.
